# Insurance, Racial/Ethnic, SES-Related Disparities in Quality of Care Among US Adults with Diabetes

**DOI:** 10.1007/s10903-013-9966-6

**Published:** 2013-12-21

**Authors:** Ruwei Hu, Leiyu Shi, Sarika Rane, Jinsheng Zhu, Chien-Chou Chen

**Affiliations:** 1 School of Public Health, and Center of Migrant Health Policy, Sun Yat-sen University, Guangzhou, China; 2 Johns Hopkins Primary Care Policy Center, Baltimore, MD USA; 3 Health Policy and Health Services Research, Department of Health Policy and Management, Johns Hopkins University Bloomberg School of Public Health, Baltimore, MD USA; 4 Department of International Business, Ling Tung University, 1, Lingtung Road, Nantun, Taichung City, 40852 Taiwan, ROC

**Keywords:** Primary care, Quality of care, Diabetes, Racial disparities

## Abstract

Diabetes-related quality improvement initiatives are typically aimed at improving outcomes and reducing complications. Studies have found that disparities in quality persist for certain racial/ethnic and socioeconomically disadvantaged groups; however, results are mixed with regard to insurance-based differences. The purpose of this study is to investigate the independent associations between type of health insurance coverage, race/ethnicity, and socioeconomic status (SES), and quality of care, as measured by benchmark indicators of diabetes-related primary care. This study used the Diabetes Care Survey of the 2010 Medical Expenditure Panel Survey. Bivariate and multivariate logistic regressions were used to examine the association between quality of diabetes care and type of insurance coverage, race/ethnicity, and SES. Multivariate analyses also controlled for additional demographic and health status characteristics. Respondents with insurance coverage (particularly those with private insurance or with Medicare and Medicaid coverage) were more likely to receive quality diabetes care than uninsured individuals. Few significant disparities based on race/ethnicity or SES persisted in subsequent multivariate analyses. Findings suggest that insurance coverage may make the greatest impact in ensuring equitable distribution of quality diabetes care, regardless of race/ethnicity or socioeconomic status. With the implementation of Affordable Care Act under which more people could potentially gain access to insurance, policymakers should next track insurance-based diabetes care disparities.

## Introduction

Diabetes is one of the leading causes of morbidity and mortality in the United States (US), and represents an enormous public health and economic burden. Nearly 26 million people in the US have diabetes—a figure that continues to climb as the population ages and chronic conditions increase in prevalence [1, 2]. As of 2010, approximately 27 % of adults ages 60 and older had diabetes, while 1.9 million adults ages 20 and older were newly diagnosed. Diabetes mellitus is the seventh leading cause of mortality in the US, and is associated with a number of health complications if preventive care and proper treatment is not received [3]. These complications can include renal disease, non-traumatic lower limb amputations, blindness, and increased risk for cardiovascular disease and stroke [1, 2, 4].

Evidence suggests that proper adherence to clinical guidelines for diabetes preventive care can reduce the risk of health complications in patients with diabetes, and improve long-term health outcomes [5]. The American Diabetes Association (ADA) established guidelines for processes of care in the preventive care of patients with diabetes; these guidelines include HbA1c testing at least twice per year, annual dilated eye examinations, and annual foot examinations [6]. In addition, the ADA and the National Cholesterol Education Program jointly established target clinical outcomes related to cardiovascular health, such as regular cholesterol testing. Meeting these targets can help patients reduce the risk of diabetic complications. For example, the ADA recommends that patients’ HbA1c levels are less than 7 %, and that total cholesterol levels are less than 200 mg/dl [7].

Despite clinical advances that have been made with regard to effective prevention, diagnosis, and treatment of diabetes, certain groups shoulder a disproportionate burden of the disease. Studies have shown that those who are poor or who comprise racial/ethnic minority groups have a higher prevalence of diabetes and are more likely to suffer diabetes-related complications that require hospitalization than their non-poor and non-minority counterparts [8–14]. For example, an estimated 13.2 % of non-Hispanic blacks and 11.9 % of Hispanics have diabetes, compared with 7.1 % for non-Hispanic whites [1, 2]. Several studies have also shown that disadvantaged groups are less likely to receive diabetes-related preventive care [15–19]. Differences in diabetes-related outcomes may be partially attributable to disparate access to care, as determined by lack of insurance or inadequate insurance coverage, having unmet needs for medical care or prescription medication, or inconsistent access to a regular source of care or primary care provider [8, 9, 20, 21]. Non-Hispanic black and Hispanic populations in particular report inconsistent access to care, and barriers to obtaining health insurance, compared with white populations [20, 21].

The literature examining disparities in quality of diabetes care is mixed. Some previous studies, primarily using state and health systems data, suggested that racial/ethnic minority populations with diabetes receive lower quality care, compared with their white counterparts [9, 11, 20–22]. However, other studies using nationally-representative datasets have shown either narrowing gaps in disparities or no significant differences in quality of diabetes care between racial/ethnic minority groups and white patients [18, 23, 24]. Although studies have examined quality of care for diabetes, few studies have compared disparities beyond those that are associated with racial/ethnic group, such as socioeconomic status or insurance coverage [25]. Among those examining insurance and diabetes care, the insured were found to receive better quality of diabetes care than the uninsured, and those who were continuously insured received better care than those who were partially insured [26–29]. However, these studies were either based on older data or conducted in only one or a few states.

The purpose of this study is to address this gap in the literature, by assessing insurance, racial/ethnic, and SES-related disparities in quality of diabetes care among US adults with diabetes. The unique contribution of this study lies in its use of a nationally-representative dataset and the inclusion of socioeconomic status (SES) in accounting for racial/ethnic and insurance-related disparities in diabetes care. The results of this study will provide up-to-date information on the topic and empirical evidence to lay the groundwork for tracking impacts of the implementation of the Affordable Care Act (ACA).

## Methods

### Data Source

Data for this study came from the 2010 Medical Expenditure Panel Survey (MEPS)—an annual nationally-representative survey of the non-institutionalized civilian population that has been administered jointly by the Agency for Healthcare Research and Quality (AHRQ) and the National Center for Health Statistics (NCHS) since 1996 [30]. The MEPS collects information from household respondents, providers, and employers in areas including health care utilization, medical expenditures, access to care, quality of care, and insurance coverage. It uses an overlapping panel design in which data are collected in five rounds of interviews over a 2.5 year period [31]. This study analyzed data from the 2010 MEPS Household Component; specifically, the Diabetes Care Survey that is periodically administered to household members who report having been diagnosed with diabetes and contains a total of nine survey items [32]. The 2010 MEPS contained a total of 32,846 observations; the current study used information from respondents aged 18 and over who completed the Diabetes Care Survey. These respondents were classified as diabetic if they reported being told by a clinician that they had diabetes. A total of 1,909 people with diabetes were included in the study, representing an estimated 20,970,670 adult population with self-reported diabetes. The overall response rate of MEPS for 2010 was 58.6 % and the response rate for the Diabetes Care Survey was 90.1 %.

### Analytical Variables

For this study, indicators of diabetes care (dependent variables) among household respondents were examined across insurance status, race/ethnicity, and SES (independent variables). Four measures of diabetes care were included in this study: (1) reported having a hemoglobin A1C measurement at least once in the past year, (2) reported having a blood cholesterol check in the past year, (3) reported having a retinal eye exam in the past year, and (4) reported having a foot examination in the past year. These measures were consistent with quality performance indicators endorsed by the National Quality Forum (NCQA) for adults aged 18–75 with diabetes [33].

Respondents’ race/ethnicity was categorized as non-Hispanic white, non-Hispanic black, Hispanic, and other. For people with multi-racial/ethnic origin, they were classified in the following order: Hispanic, black, and white. The ‘other’ category included Asians, American Indians and Alaskan Natives, and unspecified races. Since they all had relatively small sample size, they were grouped together in the analysis. Since the “other” group was so heterogenous, the results pertaining to this category were difficult to interpret and therefore not emphasized in the presentation of study findings and discussion.

Insurance status was categorized as uninsured, privately-insured, Medicare-insured, Medicaid-insured, and other publicly-insured. Categories of health insurance coverage were mutually exclusive. Persons with multiple types of health insurance were assigned to the first appropriate category in the following order: privately-insured, Medicare-insured, or Medicaid-insured, For example, the category ‘privately-insured’ included persons who had any type of private coverage either alone or in combination with other coverage. The category ‘‘Medicare-insured’’ included persons who had Medicare either alone or in combination with other public coverage. The category ‘‘Medicaid-insured’’ included persons who had Medicaid either alone or in combination with other non-Medicare, public coverage. Since the Diabetes Care Survey was a cross-sectional (one-time in 2010) survey, the insurance variable represented current status only.

SES was a composite variable comprising information from three separate measures: income, education, and employment. We used ‘poverty status’ to represent income and coded it as a dichotomous measure (1 = poor/near poor/low-income; 2 = middle income/high income). We used ‘highest education attained’ to represent education and coded it as a dichotomous measure (1 = below bachelor which includes ‘no degree,’ ‘GED,’ or ‘high school diploma’; 2 = bachelor or higher degree which includes ‘bachelor’s degree,’ ‘master’s degree,’‘doctorate degree,’ or ‘other degree’ defined in MEPS as an educational degree other than a bachelor’s, master’s, or doctorate degree.). We used ‘employment status’ to represent employment and coded it as a dichotomous measure (1 = not employed; 2 = employed). Although it was possible to have more detailed categories for these three measures, we chose to collapse into two categories per measure so that there would be enough sample (and power) per category in the combined measure of SES. The combined SES variable included four categories representing the gradient in SES, from best to worst: high-SES (with 2s from all three SES measures), above-average SES (with 2s from two of the three SES measures), below-average SES (with 2s from one of the three SES measures), and low-SES (with 1s from three SES measures).

In addition to these primary independent variables, a number of individual characteristics were measured as covariates based on their known association with use of health care services, including age (coded as a continuous measure), gender, marital status, self-reported health status (excellent/very good/good, fair/poor), limitations (i.e., self-reported impairments in activities of daily living), and geographical region (Northeast, Midwest, South, West).

### Statistical Analysis

Data analysis was performed using SAS version 9.3 [34]. Due to the complex survey design employed in MEPS, all analyses included a design effect and sampling weights in order to ensure that the sample was nationally-representative of adults living in the United States with diabetes. First, descriptive statistics were computed for all variable included in the study to yield a profile of the study sample. Next, the distribution of quality of care indicators received by adults with diabetes was obtained for each racial/ethnic group, insurance status categorization, and SES level. Chi square (χ^2^) tests were conducted to assess significant differences in quality of care across each of these three independent variables.

In addition, multivariate logistic regressions were used to examine quality of diabetes care. Models were adjusted for patients’ insurance status, race/ethnicity, SES, age, gender, marital status, health status, presence of functional limitations, and geographic region. In order to estimate these relationships, odds ratios and 95 % confidence intervals were calculated. For all analyses, a level of α < 0.05 was considered statistically significant.

## Results

### Descriptive Results

Table 1 provides descriptive results for all the variables included in the analysis. A total of 1,909 subjects with diabetes (weighted sample equals 20.9 million) were included in the analysis. Among the sampled adults with diabetes, approximately 89 % reported having a hemoglobin A1C measurement at least once in the past year, 84 % reported having a blood cholesterol check, 65 % reported having a retinal eye examination, and 69 % reported having a foot examination.

**Table 1:** Characteristics of the study sample

	Total	
	Freq	Weighted freq (%, SE)
Sample	1,909	20,970,670
Diabetes care		
Adults age 18 and over with diabetes who reported having a hemoglobin A1C measurement at least once in past year		
No	154	1,488,476 (10.33, 1.01)
Yes	1,100	12,917,698 (89.67, 1.01)
Adults age 18 and over with diabetes who reported having a blood cholesterol check in past year		
No	334	3,309,511 (16.06, 1.04)
Yes	1,538	17,293,946 (83.94, 1.04)
Adults age 18 and over with diabetes who reported having a retinal eye examination in past year		
No	721	7,287,399 (35.29, 1.36)
Yes	1,153	13,360,820 (64.71, 1.36)
Adults age 18 and over with diabetes who reported having a foot examination in past year		
No	615	6,457,397 (31.37, 1.24)
Yes	1,260	14,125,295 (68.63, 1.24)
Insurance		
Uninsured	221	1,908,648 (9.1, 0.69)
Medicaid	262	2,176,448 (10.38, 0.79)
Medicare	499	5,155,017 (24.58, 1.2)
Private	894	11,388,888 (54.31, 1.46)
Other public	33	341,669 (1.63, 0.36)
Race/ethnicity		
White	827	13,283,395 (63.34, 1.69)
Black	469	3,302,095 (15.75, 1.13)
Hispanic	442	2,913,117 (13.89, 1.23)
Other	171	1,472,062 (7.02, 0.94)
SES measures		
Poverty		
Poor/near poor/low income	862	7,545,882 (35.98, 1.51)
Middle income/high income	1,047	13,424,787 (64.02, 1.51)
Education		
Below bachelor	1,605	16,735,710 (80.26, 1.21)
Bachelor and higher degree	292	4,117,048 (19.74, 1.21)
Employment status		
Not employed	1,148	12,277,233 (58.59, 1.46)
Employed	759	8,678,267 (41.41, 1.46)
SES composite		
High SES	148	2,070,328 (9.94, 0.87)
Above-average SES	512	6,431,076 (30.86, 1.39)
Below-average SES	619	7,046,991 (33.82, 1.2)
Low SES	616	5,289,193 (25.38, 1.28)
Demographic measures		
Age^a^	1,909	60.56 (0.37)
Sex		
Male	844	10,258,465 (48.92, 1.3)
Female	1,065	10,712,204 (51.08, 1.3)
Marital status		
Not married	905	9,015,997 (42.99, 1.38)
Married	1,004	11,954,672 (57.01, 1.38)
Health status		
Excellent/VG/good	1,180	13,535,960 (64.58, 1.49)
Fair/poor	728	7,423,797 (35.42, 1.49)
Any limitation		
No	1,679	18,425,091 (87.95, 0.86)
Yes	228	2,524,808 (12.05, 0.86)
Region		
Northeast	279	3,513,472 (16.75, 1.56)
Midwest	387	4,642,834 (22.14, 1.16)
South	815	8,725,989 (41.61, 1.73)
West	428	4,088,375 (19.5, 1.22)

^a^Age results are mean (SE)

In terms of insurance status, 54.31 % had private insurance, 24.58 % had Medicare, 10.38 % had Medicaid, 1.63 % had other public insurance, but 9.1 % were uninsured. In terms of race/ethnicity, 63.34 % were non-Hispanic white, 15.75 % black, 13.89 % Hispanic, and 7.02 % other. In terms of SES, 64.02 % had middle- or high-income and 35.98 % were poor, near poor, or of low-income. For education, 19.74 % had bachelor or higher degree and 80.26 % had below-bachelor education. For employment, 41.41 % were employed and 58.59 % were unemployed (primarily due to the large proportion of the respondents who were retired). The composite SES variable showed 9.94 % categorized as high SES, 30.86 % above-average SES, 33.82 % below-average SES, and 25.38 % low SES.

In terms of demographic and health status characteristics, the mean age was 60.56, 51.08 % were females, 57.1 % were married, 64.58 % considered their health status to be excellent, very good, or good, 87.95 % had no physical limitations, and the most likely place of residence was south (41.61 %), followed by Midwest (22.14 %), west (19.5 %), and northeast (16.75 %).

### Bivariate Results

Table 2 displays the distribution of quality of care received by adults with diabetes; bivariate results are displayed by insurance type, race/ethnicity, and SES. The analysis found significant differences across insurance types for all four diabetes quality of care indicators (*p* < 0.001). Across three of the four quality indicators, adults covered by Medicare reported the highest rate of receipt of services, while adults with no insurance reported the lowest rate (89.3 vs. 57.9 % reported having a blood cholesterol check, 73.5 vs. 36.1 % reported having a retinal eye examination, and 72.3 vs. 52.1 % reported having a foot examination). Adults who were privately insured reported the highest rate of receipt of a yearly hemoglobin A1C measurement (93.02 %), and the second highest rate of receipt across the remaining three quality indicators. Uninsured adults also reported the lowest rate of receiving a yearly hemoglobin A1C measurement (73.6 %).

**Table 2:** Quality of care for US adults aged 18 and over with diabetes by insurance, race/ethnicity, and SES, MEPS 2010

	Adults age 18 and over with diabetes who reported having a hemoglobin A1C measurement at least once in past year				Adults age 18 and over with diabetes who reported having a blood cholesterol check in past year			
	n	Weighted freq	Percent	SE	n	Weighted freq	Percent	SE
Insurance (%)	1,254	14,406,175	***		1,872	20,603,457	***	
Total	1,100	12,917,698	89.67	1.01	1,538	17,293,946	83.94	1.04
Uninsured	103	943,385	73.60	3.97	134	1,090,390	57.95	3.73
Medicaid	124	1,139,937	85.90	3.00	195	1,568,187	73.12	3.12
Medicare	284	3,020,242	88.85	2.27	425	4,500,638	89.31	1.57
Private	568	7,590,485	93.02	1.16	755	9,850,705	87.98	1.25
Other public	21	223,649	93.82	4.43	29	284,024	83.13	8.96
Race/ethnicity	1,254	14,406,175	**		1,872	20,603,457	**	
Total	1,100	12,917,698	89.67	1.01	1,538	17,293,946	83.94	1.04
White	547	8,896,727	91.69	1.17	693	11,272,047	86.18	1.30
Black	230	1,685,298	85.00	2.76	368	2,572,015	79.71	2.21
Hispanic	239	1,561,417	88.82	1.93	336	2,243,624	78.76	2.41
Other	84	774,256	80.49	4.59	141	1,206,259	83.26	3.20
SES	1,245	14,320,501	*		1,858	20,470,375	**	
Total	1,094	12,859,176	89.80	1.02	1,529	17,202,227	84.03	1.05
High SES	116	1,689,937	95.51	1.94	131	1,907,421	92.92	1.88
Above-average SES	306	4,042,849	89.83	1.93	413	5,275,993	83.17	1.89
Below-average SES	350	4,255,916	90.31	1.65	502	5,810,215	83.59	1.69
Low SES	322	2,870,474	86.00	1.86	483	4,208,598	82.15	1.79

	Adults age 18 and over with diabetes who reported having a retinal eye examination in past year				Adults age 18 and over with diabetes who reported having a foot examination in past year			
	n	Weighted freq	Percent	SE	n	Weighted freq	Percent	SE
Insurance (%)	1,874	20,648,219	***		1,875	20,582,692	***	
Total	1,153	13,360,820	64.71	1.36	1,260	14,125,295	68.63	1.24
Uninsured	79	682,175	36.13	4.03	110	975,685	52.11	3.78
Medicaid	143	1,158,287	54.40	3.74	167	1,359,124	64.05	3.12
Medicare	331	3,589,891	71.27	2.35	349	3,653,910	72.60	2.38
Private	577	7,723,255	68.64	1.68	612	7,941,171	70.82	1.65
Other public	23	207,211	60.65	10.32	22	195,405	57.19	8.80
Race/ethnicity	1,874	20,648,219	***		1,875	20,582,692	*	
Total	1,153	13,360,820	64.71	1.36	1,260	14,125,295	68.63	1.24
White	532	8,921,318	68.05	1.65	560	9,142,210	70.21	1.71
Black	286	2,040,083	63.17	2.60	306	2,102,141	64.81	2.62
Hispanic	234	1,578,288	54.81	2.90	270	1,800,031	63.12	2.45
Other	101	821,131	57.49	4.86	124	1,080,913	73.74	3.70
SES	1,860	20,515,137			1,862	20,453,709		
Total	1,147	13,292,690	64.79	1.38	1,252	14,034,604	68.62	1.24
High SES	111	1,535,597	74.17	3.90	111	1,587,627	76.68	4.22
Above-average SES	293	3,997,087	62.82	2.19	339	4,248,800	67.37	2.56
Below-average SES	379	4,512,493	65.15	2.32	401	4,668,828	67.22	2.46
Low SES	364	3,247,513	62.98	2.61	401	3,529,349	68.77	1.99

* *p* < 0.05; ** *p* < 0.01; *** *p* < 0.001

Differences across racial/ethnic groups were also statistically significant for each of the four diabetes quality of care indicators. In three of the four quality of care categories, Hispanic adults reported the lowest rates of receiving diabetes services while non-Hispanic white adults reported the highest rates. With regard to having a hemoglobin A1C measurement, non-Hispanic white adults reported the highest rates (91.7 %) while those classified as ‘other’ (primarily Asians) adults reported the lowest rates (80.5 %). Among those who had blood cholesterol checked in the past year, non-Hispanic white adults reported the highest rate (86.2 %) while Hispanic adults reported the lowest rate (78.8 %). Rates of yearly retinal eye examinations were highest among non-Hispanic white adults (68 %) and lowest among Hispanic adults (54.8 %). Rates of yearly foot examinations were highest among ‘other’ adults with diabetes (73.7 %) and lowest among Hispanic adults (63.1 %).

With regard to differences across SES categories, bivariate findings were statistically significant for receipt of a hemoglobin A1C measurement (*p* < 0.05) and a blood cholesterol check in the past year (*p* < 0.01). The breakdown of results was unsurprising—across all four diabetes quality of care indicators, adults in the high SES category reported the highest rates of receipt while adults with low SES reported the lowest rates.

### Multivariate Results

Table 3 presents the results of multivariate logistic regression models that explored the association between diabetes quality of care and health insurance, race/ethnicity, and SES, while adjusting for age, gender, marital status, perceived health status, functional limitations, and census region.

**Table 3:** Logistic regressions of quality of care for US adults aged 18 and over with diabetes, MEPS 2010

	Odds ratio (95 % CI)			
	Adults age 18 and over with diabetes who reported having a hemoglobin A1C measurement at least once in past year	Adults age 18 and over with diabetes who reported having a blood cholesterol check in past year	Adults age 18 and over with diabetes who reported having a retinal eye examination in past year	Adults age 18 and over with diabetes who reported having a foot examination in past year
Insurance				
Uninsured	0.205*** (0.105 0.401)	0.259*** (0.165 0.408)	0.383*** (0.249 0.59)	0.577** (0.391 0.851)
Medicaid	0.494 (0.231 1.059)	0.502** (0.308 0.819)	0.868 (0.548 1.375)	0.946 (0.63 1.421)
Medicare	0.679 (0.341 1.354)	0.765 (0.474 1.235)	0.882 (0.632 1.232)	0.888 (0.643 1.227)
Other	1.557 (0.33 7.338)	0.95 (0.259 3.489)	1.019 (0.404 2.569)	0.748 (0.358 1.56)
Private (reference)	1	1	1	1
Race/ethnicity				
Black	0.538** (0.337 0.857)	0.872 (0.608 1.25)	1.115 (0.845 1.471)	0.933 (0.693 1.256)
Hispanic	1.236 (0.696 2.195)	1.026 (0.654 1.61)	0.792 (0.578 1.085)	1.099 (0.805 1.501)
Other	0.417** (0.223 0.779)	0.989 (0.558 1.756)	0.651 (0.409 1.035)	1.5 (0.971 2.317)
White (reference)	1	1	1	1
SES				
High SES	2.977* (1.016 8.724)	2.567** (1.254 5.254)	1.548 (0.932 2.573)	1.695 (0.993 2.894)
Above-average SES	1.215 (0.616 2.397)	0.847 (0.538 1.333)	0.754 (0.532 1.07)	0.984 (0.677 1.428)
Below-average SES	1.462 (0.863 2.476)	0.941 (0.67 1.323)	0.92 (0.679 1.248)	0.911 (0.68 1.221)
Low SES (reference)	1	1	1	1
Age	0.999 (0.976 1.022)	1.03*** (1.017 1.042)	1.029*** (1.019 1.04)	1.019*** (1.008 1.03)
Sex				
Female	1.312 (0.843 2.042)	1.207 (0.908 1.605)	1.347* (1.067 1.701)	0.834 (0.634 1.097)
Male (reference)	1	1	1	1
Marital status				
Not married	0.949 (0.573 1.569)	0.723 (0.508 1.03)	0.511*** (0.395 0.66)	0.845 (0.65 1.097)
Married (reference)	1	1	1	1
Health status				
Fair/poor	1.757* (1.049 2.942)	1.189 (0.848 1.666)	0.839 (0.642 1.095)	1.129 (0.872 1.463)
Excellent/VG/Good (reference)	1	1	1	1
Any limitation				
Yes	0.958 (0.39 2.354)	1.566 (0.928 2.644)	0.818 (0.567 1.18)	2.146*** (1.512 3.047)
No (reference)	1	1	1	1
Region				
Northeast	0.955 (0.486 1.875)	1.892* (1.147 3.121)	2.63*** (1.836 3.767)	1.298 (0.838 2.009)
Midwest	1.473 (0.823 2.637)	1.573* (1.052 2.353)	1.357* (1.014 1.817)	1.325 (0.983 1.784)
West	0.701 (0.39 1.259)	1.266 (0.837 1.914)	1.368 (0.984 1.904)	0.741* (0.558 0.986)
South (reference)	1	1	1	1

* *p* < 0.05; ** *p* < 0.01; *** *p* < 0.001

The analyses did find significant insurance-based disparities in quality of care indicators; respondents who were uninsured had lower odds of receiving diabetes services than privately insured patients, but there were few differences between privately insured patients and those with Medicaid, Medicare, or other public insurance. For example, compared to the privately insured, the uninsured had significantly lower odds of reporting having a hemoglobin A1C measurement in the past year (0.205, *p* < 0.001), having a blood cholesterol check (0.259, *p* < 0.001), having a retinal eye examination (0.383, *p* < 0.001), and having a foot examination (0.577, *p* < 0.01). Similar relationships were found between the uninsured and Medicare-insured (results not shown, but available upon request). Among the insured, only Medicaid-insured had lower odds of reporting having a blood cholesterol check in the past year when compared to the privately insured (0.502, *p* < 0.01).

The analyses found racial/ethnic disparities persisted in diabetes quality of care in only one of the four measures. Both non-Hispanic black and other minority adults had lower odds of reporting receiving a hemoglobin A1C measurement at least once in the past year compared with non-Hispanic white adults (*p* < 0.01).

The analyses also found some SES disparities on selected diabetes quality of care measures. Specifically, compared to the low SES group, the high SES group had significantly greater odds of reporting having a hemoglobin A1C measurement in the past year (2.977, *p* < 0.05) and having a blood cholesterol check (2.567, *p* < 0.01).

## Discussion

This study used the most recently available MEPS data to explore the presence of disparities in quality of diabetes care, and to build on past research investigating whether racial/ethnic and sociodemographic differences in quality of care persist despite the implementation of quality improvement initiatives across the US [35, 36]. Based on the unadjusted results, we found that insurance coverage and race/ethnicity were significantly associated with having each of the four benchmark diabetes quality of care indicators within the past year—a hemoglobin A1C measurement, a blood cholesterol check, a retinal eye examination, and a foot examination. Similarly, SES was significantly associated with having a hemoglobin A1C measurement and having a blood cholesterol check.

After controlling for other demographic and health status characteristics, we found that the significant association between insurance coverage and quality of care remained. In other words, respondents who had some form of insurance (i.e., private insurance, Medicare, or Medicaid) were more likely to receive diabetes services than uninsured respondents across all four quality of care indicators. In contrast, most of the significant differences across quality of care based on race/ethnicity or SES level disappeared in the multivariate analyses—findings that are consistent with previous studies that have shown narrowing or no disparities based on race/ethnicity [18, 24, 37].

Our findings suggest that insurance coverage, rather than race/ethnicity or socioeconomic status, makes the greatest difference for ensuring that diabetes care meets minimum quality of care medical standards. These findings corroborate studies in the literature that point to insurance as an important factor in mitigating health and health care disparities between those groups who are socially disadvantaged versus others [25, 38]. Although some racial/ethnic and SES disparities in quality appear to persist, this study suggests that an expansion of different types of insurance coverage would make the biggest impact in terms of improving the quality of diabetes care and reducing the disproportionate burden of the disease and its complications.

Traditionally, public health practitioners used education campaigns to inform the public about health threats and how to avoid them. However, recent legislation and government regulation activities established a national framework that incorporates the obesity prevention goals and supports the use of policy and law to change social norms and reverse the obesity epidemic [39]. For example, the Patient Protection and ACA is an important step undertaken by the federal government to address health disparities. The legislation included provisions to expand prescription drug coverage for those covered under Medicare or other health plans. Furthermore, it contains a number of provisions aimed at reducing barriers to access for medically-underserved and disadvantaged populations, such as expanded access to primary and preventive care services through community health centers, premium and cost-sharing subsidies, and Medicaid expansions to provide coverage for more low-income individuals and families. In addition to the ACA, performance-based coverage models such as Accountable Care Organizations and Patient-centered Medical Home (PCMH) could make a positive impact to ensure that those with diabetes receive services that are proven to optimize health outcomes [40].

We must note several limitations of this study. First, study results are based on self-reported information from adult respondents and subject to recall bias and subsequent threats to reliability. Second the analysis was based on cross-sectional data, which only allows us to draw inferences of association rather than causation. However, our conclusions are strengthened by the strong survey design and nationally-representative sample of responses included in the analysis. Finally, this study examined diabetes quality of care based on four benchmark indicators of services received by respondents. It would have been useful to include variables that captured additional information on health care use and health status of respondents, such as accessibility of care, type of provider seen, frequency of health care use, or disease severity. Future analyses could be strengthened by such factors; however, our inclusion of variables capturing self-reported health status and functional limitations helped mitigate this limitation.

In conclusion, this study strongly suggests that health insurance plays a large role in ensuring quality diabetes care and mitigating disparities based on race/ethnicity or socioeconomic status. Many quality improvement initiatives aimed at improving health outcomes are intended to address disparities by focusing on chronic illness such as diabetes—a strategy based on the fact that traditionally disadvantaged and underserved populations disproportionately carry the burden of diabetes and its complications [35, 41]. However, health care providers and policymakers must recognize expanded or enhanced insurance coverage as an effective tactic in addressing barriers to accessing care, and improving the quality diabetes care and health outcomes. The ACA is moving towards the right direction as a legislation towards expanding insurance coverage and creating a regulatory engine that will work to move the US from a system that follows the customary care model of medical care toward an evidence-based system of medical care [42, 43], but great efforts need also be given to enhance quality care to those insured regardless of type of insurance coverage.
